# Three-year clinical and radiological results of a cruciate-retaining type of the knee prosthesis with anatomical geometry developed in Japan

**DOI:** 10.1186/s12891-021-04114-x

**Published:** 2021-03-03

**Authors:** Arata Nakajima, Manabu Yamada, Masato Sonobe, Yorikazu Akatsu, Masahiko Saito, Keiichiro Yamamoto, Junya Saito, Masaki Norimoto, Keita Koyama, Hiroshi Takahashi, Yasuchika Aoki, Toru Suguro, Koichi Nakagawa

**Affiliations:** 1 Department of Orthopaedic Surgery, Toho University Sakura Medical Center, 564-1 Shimoshizu, Sakura, Chiba, 285-8741 Japan; 2 Department of Orthopaedic Surgery, Chiba Medical Center, 1-7-1 Minami-cho, Chuo-ku, Chiba, 261-0013 Japan; 3 Department of Orthopaedic Surgery, Faculty of Medicine, University of Tsukuba, 1-1-1 Tennodai, Tsukuba, Ibaraki 305-8575 Japan; 4 Department of General Medical Sciences, Graduate School of Medicine, Chiba University, 1-8-1 Inohana, Chuo-ku, Chiba, 260-8677 Japan; 5 Department of Orthopaedic Surgery, Eastern Chiba Medical Center, 3-6-2 Okayamadai, Togane, Chiba, 283-8686 Japan; 6 Japan Research Institute of Artificial Joint, 725-1 Sugo, Kisarazu, Chiba, 292-0036 Japan

**Keywords:** FINE total knee, Cruciate-retaining (CR), Total knee arthroplasty (TKA), Patient-reported outcomes (PROs)

## Abstract

**Background:**

The FINE total knee was developed in Japan and clinical use began in 2001. It has unique design features, including an oblique 3^o^ femorotibial joint line that reproduces anatomical geometry. Although 20 years have passed since the FINE knee was clinically used for the first time in Japan, a formal clinical evaluation including patient-reported and radiographic outcomes has not been undertaken.

**Methods:**

A total of 175 consecutive primary cruciate-retaining (CR)-FINE total knee arthroplasties (TKAs) at our hospital between February 2015 and March 2017 were included in this study. Three years postoperatively, range of motion (ROM), Knee Society Score (KSS), Knee Injury and Osteoarthritis Outcome Score (KOOS) and Forgotten Joint Score (FJS) were recorded and compared with preoperative scores. Radiographic analyses including mechanical alignment, component alignment, and incidence of radiolucent lines also were undertaken based on the radiographs 3 years postoperatively.

**Results:**

One-hundred twenty-two knees (70%) were available for 3-year follow-up data using KOOS, except for the sports subscale. Postoperative KOOS-symptom, −pain and -ADL were > 85 points, but KOOS-sports, −QOL and FJS were less satisfactory. ROM, KSS and all the subscales of KOOS were significantly improved compared with preoperative scores. Postoperative mean FJS was 66 and was significantly correlated with all the subscales of KOOS, but not with postoperative ROM. Radiolucent lines ≧1 mm wide were detected in five knees (4.1%). There were no major complications needing revision surgeries.

**Conclusions:**

Patient-reported outcomes (PROs) for symptoms, pain and ADL after the CR-FINE TKA were generally improved, but those for sports, QOL and FJS were improved less. The incidence of radiolucent lines was rare but detected around the femoral components. With the mid- to long-term follow-up, improvements of surgical technique will be necessary to achieve better PROs from patients receiving the FINE knee.

## Background

Although the outcomes of total knee arthroplasty (TKA) are generally acceptable, approximately 20% of patients have some complaints after TKA [1–3]. The reasons for dissatisfaction after TKA remain poorly understood; however, failure of restoration of a physiological joint line has been suggested as a causative factor. In 2011, Bellemans introduced kinematically aligned (KA)-TKA as a surgical technique to realize a physiological joint line [4]. The goal of KA-TKA is to maintain the orientation of the native joint line. While there have been studies showing that KA-TKA provides equivalent or better function and similar survival rate to mechanically aligned (MA)-TKA [5–10], the longevity of polyethylene inserts and femoral and/or tibial components implanted not perpendicular to the mechanical axis are a concern [11–13]. However, as a concise follow-up at 20 years of modern TKA with cement reported that neutral mechanical alignment did not provide better implant survivorship than the outlier group [14], it remains unknown whether KA-TKA provides better clinical outcomes and survivorship than MA-TKA.

**Fig. 1:**
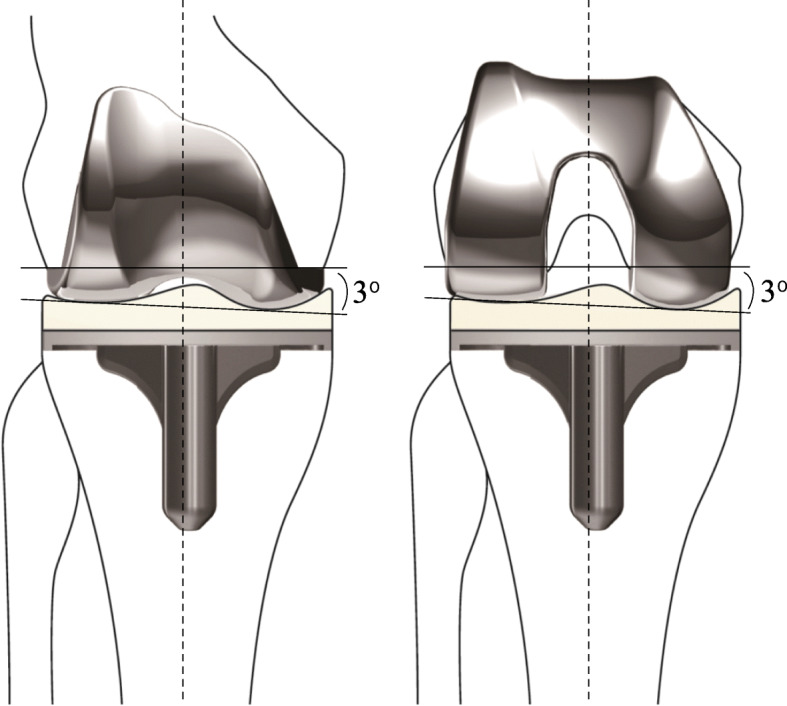
The FINE total knee. The femoral condyle has an asymmetric shape and femorotibial joint line with an oblique 3^o^ both in coronal (left) and axial (right) planes which is incorporated into the implant design. The medial surface of the polyethylene insert has a convex curve while the lateral surface possesses a flat surface. FINE reproduces anatomical geometry by conducting osteotomy perpendicular to the mechanical axis. The figure is reprinted with minor modifications from Fig. 1 in the reference no. [15]

The FINE total knee has been developed in Japan and used for approximately 20,000 TKAs of Japanese patients since 2001. It has unique design features, including an oblique 3^o^ femorotibial joint line (Fig. 1). This feature allows to reproduce anatomical geometry by cutting the bone perpendicular to the mechanical axis. The sagittal curvature of the femoral component has dual-radii in extension and flexion range. The polyethylene insert also has a unique design; the medial surface has a convex curve with increased conformity to the femoral component while the lateral has a flat surface. These features of the FINE knee allow KA-TKA via conventional osteotomy, and enhance internal rotation of the tibia and femoral rollback via medial pivot motion [15], which would expect better patient-reported outcomes.

Although 20 years have passed since this implant was clinically used for the first time in Japan, a formal clinical evaluation including patient-reported and radiographic outcomes has not been undertaken. The aim of the present study was to evaluate the 3-year clinical results including patient-reported and radiographic outcomes in Japanese patients receiving a cruciate-retaining (CR) type of the FINE total knee.

## Methods

### Patients

**Fig. 2:**
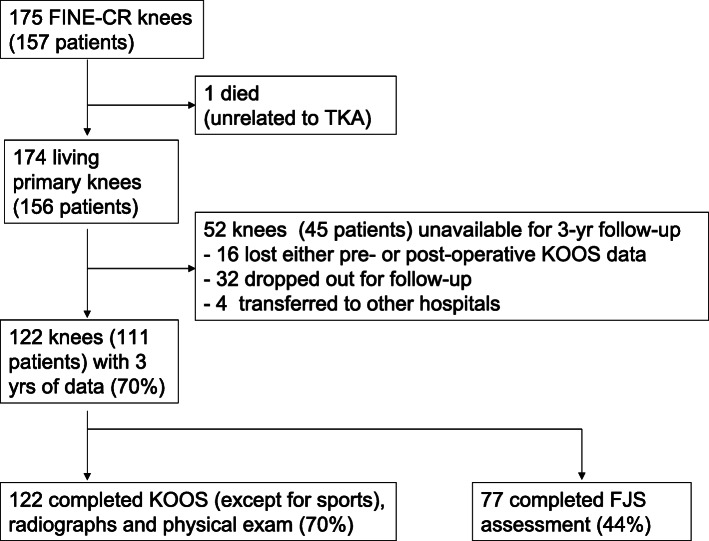
A flowchart of the patients during this study. TKA, total knee arthroplasty; KOOS, Knee Injury and Osteoarthritis Outcome Scores; FJS, Forgotten Joint Score

A total of 175 consecutive primary TKAs using a CR type of the FINE total knee (Teijin-Nakashima Medical Co. Ltd., Okayama, Japan) in 157 patients at our hospital between February 2015 and March 2017 were included in this study. One patient (one TKA) died from TKA-unrelated causes. None received a revision. Of the remaining 174 TKAs (156 patients), 122 knees (111 patients, 70%) were available for 3-year follow-up data; the data for the Knee Injury and Osteoarthritis Outcome Score (KOOS) [16] except for a sports subscale and radiographs were available for all of those while data for KOOS-sports and the Forgotten Joint Score (FJS) [17] were available for 53 (30%) and 77 knees (44%), respectively (Fig. 2). There were nine men and 102 women, with a mean age of 72.3 years (29–89) at the time of surgery. The mean body mass index was 27.2 kg/m^2^ (16.7–39.6). One hundred twelve TKAs were performed for osteoarthritis, seven for rheumatoid arthritis (RA), and three for osteonecrosis (Table 1).

**Table 1:** Summary of demographic data for patients included in this study

Mean age at surgery, yrs. (range)	72.3 (29–89)
Gender, female/male	102/9
Disease, OA/RA/ON	112/7/3
Mean height at surgery (range)	153 (136–173)
Mean weight at surgery (range)	63.4 (33.9–97.5)
Mean BMI at surgery (range)	27.2 (16.7–39.6)
Mean preoperative standing FTA, degree (range)	185 (152–209)
Mean preoperative HKA angle (range)	11.2 (−20 to 35)
Mean operation time, min (range)	125 (89–185)
Patellar replacement, yes/no	80/42

*OA* osteoarthritis, *RA* rheumatoid arthritis, *ON* osteonecrosis, *BMI* body mass index, *FTA* femorotibial angle, *HKA* hip-knee-ankle. Varus mechanical alignment is designated as positive for HKA angle

This study was approved by the institutional review board at our institution (approved number: S17017). All activities were performed in accordance with the ethical standards set forth in the Declaration of Helsinki, and informed consent was obtained from all patients who participated in this study.

### Surgical procedures

All TKAs were performed using the measured resection technique by anterior reference [18]. Surgical approaches were chosen either mid-vastus or sub-vastus for varus knees, but the lateral parapatellar approach was used for valgus knees. A release of the deep fibers of the medial collateral ligament (MCL) was routinely performed for varus knees. Surgeries were performed using conventional instruments; that is, distal femoral osteotomy was conducted perpendicular to the mechanical axis at a level 9–10 mm from the farthest point of the medial condyle, and the posterior condyle was osteotomized parallel to the surgical epicondylar axis (3°external to the posterior condylar line). A tibial osteotomy subsequently was conducted perpendicular to the anatomical axis of the tibia. The cutting level was set 8–10 mm distal to the convex of the lateral plateau. Following osteotomy, adjustments for soft tissue balancing were performed before the implants were fixed to the bone with cement. Whether to replace the patellar component or not depended on the surgeons’ decision; 80 knees received patellar replacement (66%, Table 1). Patients were discharged 3 weeks after surgery when they were medically stable, with pain controlled by oral analgesics and deemed by a physiotherapist to be mobilizing sufficiently to function safely at home.

### Radiographic examinations

**Fig. 3:**
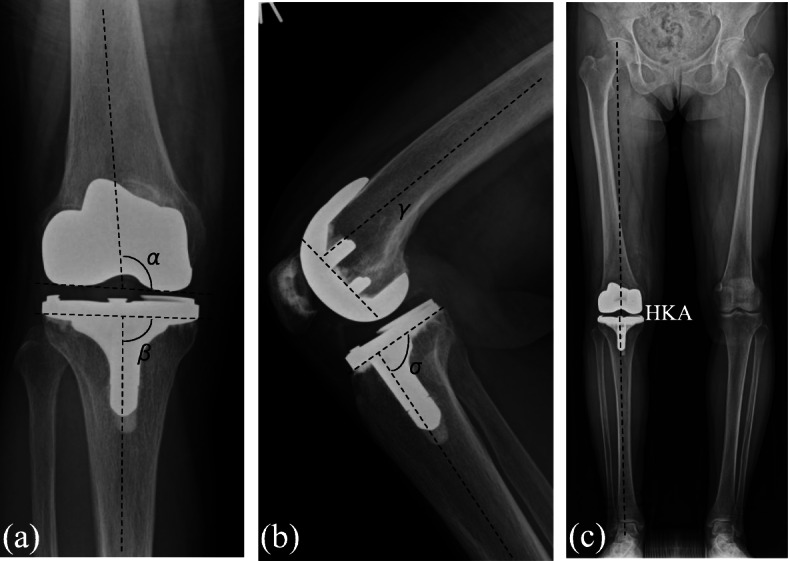
Representative (**a**) anteroposterior radiograph of a CR-FINE TKA showing measurement of the distal femoral valgus angle (DFVA; α) and proximal tibia varus angle (PTVA, β). **b** Lateral radiograph showing measurement of the femoral flexion angle (FFA; γ) and tibial slope (TS; σ). **c** Full-length standing radiograph showing measurement of the hip-knee-ankle (HKA) angle. Varus mechanical alignment is designated as positive for HKA angle

Routine postoperative assessment included anteroposterior, lateral, and 60°skyline radiographs of the knee, and full-length standing radiographs of both lower limbs. The anatomical axis (the angle subtended by lines bisecting the medullary canals of the femur and the tibia) and the mechanical axis (the angle subtended by lines connecting the center of the femoral head and the center of the femoral condyles, and the center of the tibial plateau to the center of the talus) were measured from full-length standing radiographs. The alignment of the components was assessed on AP radiographs of the knee using the distal femoral valgus angle (DFVA, α) and proximal tibial varus angle (PTVA, β), while the femoral flexion angle (FFA, γ) and tibial slope (TS, σ) were measured on lateral radiographs. The mechanical alignment was assessed by the hip-knee-ankle (HKA) angle based on the full-length standing radiographs with varus alignment designated as positive (Fig. 3). These measurements were performed using the protocol of Kilincoglu et al. [19]. Three independent observers (AN, MY, KY) examined radiographs for evidence of anterior notching of the femur, component failure or subsidence, lucent lines, osteolysis, and heterotopic ossification based on the standardized Knee Society radiological evaluation system [20].

### Clinical evaluation

We used the Knee Society Score (KSS), which consists of a knee score (KSS-KS) and a function score (KSS-FS), as an objective evaluation of knee function [21]. In addition to the KSS, we used the Japanese KOOS, an instrument of confirmed validity and reliability for patient-reported outcomes (PROs) based on its cross-cultural adaptation [22]. The KOOS consists of a total of 42 knee-related items, and each item was scored from 0 to 4. Five KOOS subscales, including symptoms (KOOS-symptom), pain (KOOS-pain), ADL (KOOS-ADL), sports/recreation (KOOS-sports), and quality of life (KOOS-QOL) were converted to 100 points [16]. Furthermore, we investigated the FJS for 77 TKAs (72 patients). The range of motion (ROM) was measured using a goniometer. Postoperative scores were compared with the preoperative scores. Both intraoperative and postoperative complications were noted from the medical records.

### Statistical analysis

The reliability of each radiographic measurement was assessed using intraclass correlation coefficients. All radiographic measurements in this study showed good reliability (all values > 0.8), and discrepancies were discussed until consensus was achieved. The paired t-test was used to compare 3-year postoperative with preoperative scores. Results were expressed as the mean (standard deviation, SD). Correlations among postoperative KSS, KOOS and FJS were analyzed by Pearson’s correlation coefficients. Data analyses were performed using SPSS software, version 21 (SPSS Inc., Chicago, IL, USA) and *p*-values of < 0.05 were considered statistically significant.

## Results

### Clinical outcome scores

Mean postoperative KSS-KS, KSS-FS, KOOS subscales, and FJS were shown in Tables 2 and 3. Patients reported clinically and statistically significant improvements in the KSS-KS, KSS-FS, and all subscales of the KOOS (*p* < 0.001).

**Table 2:** Summary of ROM and KSS

	Preoperative	Postoperative 3-year	P-value
ROM, degree (SD)	110 (19.8)	123 (14.4)	< 0.001
KSS-KS (SD)	43.5 (14.5)	97.2 (4.90)	< 0.001
KSS-FS (SD)	40.0 (19.5)	76.0 (18.8)	< 0.001
Extension, degree (SD)	−10.1 (9.94)	−0.78 (3.21)	< 0.001
Flexion, degree (SD)	120 (14.3)	124 (13.3)	0.003

*ROM* range of motion, *KSS* Knee Society Score, *KS* Knee score, *FS* Function score, *SD* standard deviation

**Table 3:** Summary of patient-reported outcomes (PROs)

	Preoperative	Postoperative 3-year	P-value
Mean KOOS-symptom (SD)	48.0 (18.9)	85.3 (13.7)	< 0.001
Mean KOOS-pain (SD)	42.3 (18.4)	89.4 (12.8)	< 0.001
Mean KOOS-ADL (SD)	58.5 (16.1)	85.7 (13.3)	< 0.001
Mean KOOS-sports (SD)	21.1 (15.1)	53.5 (28.7)	< 0.001
Mean KOOS-QOL (SD)	26.1 (14.3)	70.5 (20.7)	< 0.001
Mean FJS (SD)	–	66.2 (23.8)	–

*KOOS* Knee Injury and Osteoarthritis Outcome Score, *FJS* Forgotten Joint Score, *SD* standard deviation

The postoperative flexion angle increased significantly compared with the preoperative one (*p* = 0.003, Table 2). Seven knees had limited extension; six knees had an extension limitation between 10°and 20°and one knee had a limitation of 25°.

### Correlations among postoperative KSS, KOOS and FJS

Correlations among postoperative KSS, KOOS and FJS were computed to investigate the relationships among these postoperative outcomes. FJS was correlated significantly with KSS-FS and all the subscales of KOOS, but not with postoperative flexion angle or ROM (Table 4).

**Table 4:** Correlations among postoperative KSS, KOOS and FJS

	Extension	Flexion	ROM	KSS-KS	KSS-FS	KOOS-symptom	KOOS-pain	KOOS-ADL	KOOS-sports	KOOS-QOL	FJS
Extension		−0.230*	−0.436**	− 0.325**	−0.169	− 0.145	−0.068	− 0.187*	−0.310*	− 0.175	−0.177
Flexion			0.976**	0.465**	0.097	−0.086	−0.031	0.055	0.086	−0.019	−0.016
ROM				0.503**	0.127	−0.047	−0.014	0.092	0.159	0.021	0.007
KSS-KS					0.165	0.235**	0.451**	0.385**	0.319*	0.294**	0.289
KSS-FS						0.202*	0.212*	0.432**	0.579**	0.362**	0.240*
KOOS-symptom							0.671**	0.614**	0.367**	0.539**	0.563**
KOOS-pain								0.739**	0.447**	0.561**	0.596**
KOOS-ADL									0.657**	0.642**	0.653**
KOOS-sports										0.619**	0.344*
KOOS-QOL											0.633**
FJS											

*ROM* range of motion, *KSS* Knee Society Score, *KS* Knee score, *FS* Function score, *KOOS* Knee Injury and Osteoarthritis Outcome Score, *FJS* Forgotten Joint Score. *P < 0.05, **P < 0.01

### Radiographic outcomes

Mean postoperative standing FTA, DFVA (α), PTVA (β), FFA (γ) and TS (σ) are shown in Table 5. Mean postoperative HKA angle (SD) was 2.3 (3.6)° with slightly varus alignment, and 74 knees (60.7%) were within ±3° of the HKA angle.

**Table 5:** Radiographic analysis for CR-FINE TKA 3 years postoperatively

Component alignment	
Mean standing FTA (SD), degree	176 (2.8)
Mean DFVA (SD), degree	98 (2.4)
Mean PTVA (SD), degree	89 (1.9)
Mean FFA (SD), degree	2.5 (2.1)
Mean TS (SD), degree	87 (1.6)
Mechanical alignment	n (%)
Neutral (HKA angle = 0°)	12 (9.8)
Varus (HKA angle≧1°)	88 (72.1)
Valgus (HKA angle≦ − 1°)	22 (18.0)
Within HKA angle ±3°	74 (60.7)
Mean HKA angle (SD), degree	2.3 (3.6)
Periprosthetic bone reaction	n (%)
Radiolucent lines (≧1 mm)	
femur	5 (4.1)
tibia	0
Osteolysis	0
Subsidence	0
Heterotopic ossification	1 (0.8)

*FTA* femorotibial angle, *DFVA* distal femoral valgus angle, *PTVA* proximal tibial varus angle, *FFA* femoral flexion angle, *TS* tibial slope, *HKA* hip-knee-ankle, *SD* standard deviation. Varus mechanical alignment is designated as positive for HKA angle

Radiographic analyses 3 years postoperatively revealed no instances of osteolysis or subsidence. Radiolucent lines ≧1 mm wide were detected in five knees (4.1%), all of which occurred in zone 4 of the femoral components [20] but were insignificant clinically. There was one knee with heterotopic ossification in the quadriceps (0.8%), but it was asymptomatic (Table 5).

### Complications

One patient died due to a cause unrelated to TKA. One had a suspicious deep infection but joint fluid culture was negative for bacteria and the knee was not revised. There were eight partial tears of the popliteal tendon intraoperatively, all of which were sutured using nylon thread. One patient had an intraoperative avulsion of the superficial fibers of the MCL from its insertion to the tibia, which was reconstructed by suture and pull-out. One patient with RA had a medial subchondral fracture of the proximal tibia intraoperatively, which was fixed using a cancellous screw. There was one lateral supracondylar fracture intraoperatively in an RA patient and one anterior femoral notching without a periprosthetic fracture, but no additional surgeries were required. One patient with a severely deformed valgus knee (preoperative femorotibial angle: 152°) had transient peroneal nerve palsy postoperatively but had recovered fully by the 3-year follow-up (Table 6).

**Table 6:** Summary of intra- and post-operative complications in 3 years after CR-FINE TKA

Complications	n (%)	Notes
**Major complications**		
Suspicious deep infection	1 (0.8)	No bacteria detected, not revised
**Minor complications**		
Anterior notching	4 (3.3)	No fracture
Popliteus tendon tear	5 (4.1)	All sutured
MCL tear	1 (0.8)	Sutured
Lateral epicondyle fracture	1 (0.8)	
Peroneal nerve palsy	1 (0.8)	Full recovery

*MCL* medial collateral ligament

## Discussion

The most characteristic point of the FINE knee is the design that reproduces the anatomical geometry, that is, a 3°obliquity built into the medial femorotibial surface in both coronal and axial planes. Here we showed that the postoperative mean DFVA was 98^o^, slightly more than that of the conventional prostheses. These features allow surgeons to perform KA-TKA by cutting the bone perpendicular to the mechanical axis. Furthermore, the FINE knee adopts an ultra-high molecular weight polyethylene insert including vitamin E with antioxidant properties. As there is an increased risk of revision in KA-TKA using conventional prostheses [23, 24], these characteristic designs of the FINE knee are expected to show superior longevity to the conventional prostheses implanted in kinematic alignment.

The second characteristic point of the FINE knee is the polyethylene insert, which is dish-shaped medially and has a flat-surface laterally. This structure allows natural internal rotation of the tibia and roll-back of the lateral femoral condyle during flexion, leading to deep flexion. Here, the mean postoperative flexion angle (SD) was 124 (13)° (Table 2). The correlation analyses among postoperative flexion angle, KSS, KOOS and FJS showed significant correlations between flexion angle and KSS-KS, while flexion angle did not show any significant correlations with KOOS subscales or FJS (Table 4). These results suggest that postoperative flexion angle has a significant impact on a physician-based objective evaluation but not on PROs.

For all patients included in this study, we performed TKAs using a measured resection (MR) technique. Van Lieshout et al. showed that the joint line was elevated after TKA using the MR technique [25]. In addition, Luyckx et al., using cadaver knees, demonstrated that despite a well-balanced knee in full extension and at 90°of flexion, increased mid-flexion instability was evident in knees in which the joint line was raised [26]. Because we cut femoral posterior condyles by anterior reference and used a CR-type for the patients who participated in this study, we might have implanted femoral components that were smaller than the anatomical anteroposterior length of the femoral condyles, and this may have caused shortening of the posterior condylar offsets. This may also raise the joint line, which in turn, causes mid-flexion instability.

Recently, the FJS has been used to evaluate top-performing TKAs since it has a diminished ceiling effect [17]. Parratte et al. described a cohort of posterior-stabilized Zimmer LPS-Flex TKA (Zimmer Biomet, Warsaw, Indiana) with a mean FJS of 74 at a mean follow-up of 3.8 years [27], while Thomsen et al. reported mean FJS for the Vanguard CR TKA (Zimmer Biomet) of between 44 and 59 from 1 to 4 years following mobile-bearing or CR TKAs [28]. Moreover, excellent 5-year clinical results of the medial ball and socket TKAs were demonstrated by Katchky et al. [29]. According to their report, the mean postoperative flexion angle was 124°. It was surprising that postoperative KOOS-symptom, −pain and -ADL were > 90, with KOOS-sports being 71 and KOOS-QOL being 82. Furthermore, postoperative Oxford Knee Score and FJS were 44 (full score: 48) and 75, respectively. These results are better than the results obtained from patients receiving a CR-type of the FINE knee. Although a simple comparison between the two different prostheses should be avoided as the backgrounds of the patients were different, these excellent mid-term results of the medial ball and socket knee suggest the importance of the congruent medial articulation.

To obtain better PROs from the FINE knee, we are now performing TKAs using the pre-cut technique developed by Kaneyama et al. [30], which creates the extension gap first and then a small temporary gap in flexion before the final cut of the femoral posterior condyles and allows to measure the flexion gap easily with the PCL intact. Using this technique, surgeons can determine the amount of additional cutting of the femoral posterior condyles to the extension gap to be adjusted. Adjustment of the flexion gap to the extension gap is expected to avoid shortening of the posterior condylar offset, leading to better ROM and stability in flexion after TKA [31–33]. Although we could not show better PROs for the CR-FINE knee than other top-performing prostheses developed in the US or Western Europe, adjustment of the flexion gap to the extension gap using the pre-cut technique will raise the PROs after the CR-FINE TKA. This is required to be addressed by the future clinical studies.

Radiographic analyses for the CR-FINE TKA 3 years postoperatively demonstrated radiolucent lines more than 1 mm wide in five knees (4.1%) (Table 6). All radiolucent lines were detected in zone 4 on the femoral component side. The possible reason for the radiolucent line in this location may be the application of less or no bone cement in that zone to avoid spill-over after implantation of the femoral components. Although no clinical problems occurred over 3 years postoperatively, continuous attention should be paid to whether these radiolucent lines will spread or not. It should be noted that no subsidence of the tibial components occurred during the 3-year follow-up period. Tibial base plates of the FINE knee adopt an asymmetric design between medial and lateral sides, which provides good coverage on the cut surface of the tibia. These features probably contribute to the absence of subsidence of the tibial components.

There are some limitations to this study. First, the sample size was relatively small and the patients were recruited from a single institution. Second, 30% of the knees were not available for analysis of 3-year KOOS data. Moreover, only 30% of knees were available for a KOOS-sports subscale, and 44% were available for FJS. Nevertheless, our results show that the FINE knee is widely acceptable for Japanese patients with knee deformities, considering the good PROs comparable to other top-performing knee prostheses.

## Conclusions

We showed 3-year clinical results of a CR-type of the FINE total knee that was mechanically aligned through the MR technique. Postoperative KOOS-symptom, −pain and -ADL were > 85 points, but KOOS-sports, −QOL and FJS were less satisfactory. There were no major complications needing revision surgeries. Incidence of radiolucent lines was rare around the femoral components. There were no instances of osteolysis or subsidence. With the mid- to long-term follow-up, improvements of surgical technique will be necessary to achieve better PROs from patients receiving the FINE knee.

## Data Availability

All data generated or analyzed during the current study are included in this published article.

## Abbreviations

CR: Cruciate-retaining
TKA: Total knee arthroplasty
KSS: Knee Society Score
KOOS: Knee injury Osteoarthritis Outcome Score
PROs: Patient-reported outcomes
FJS: Forgotten Joint Score
KA: Kinematic alignment
ROM: Range of motion
FTA: Femorotibial angle
HKA: Hip-knee-ankle
DFVA: Distal femoral valgus angle
PTVA: Proximal tibial varus angle
FFA: Femoral flexion angle
TS: Tibial slope
